# Hyponatremic Seizures and Adrenal Hypoplasia Congenita in a Neonate with Congenital Diaphragmatic Hernia

**DOI:** 10.1155/2019/4178251

**Published:** 2019-05-27

**Authors:** Sourabh Verma, Sheryl Purrier, Emily Breidbart, John G. Pappas, Pradeep V. Mally, Tara M. Randis

**Affiliations:** ^1^Division of Neonatology, Department of Pediatrics, New York University School of Medicine, New York, NY, USA; ^2^Division of Pediatric Endocrinology, Department of Pediatrics, New York University School of Medicine, New York, NY, USA; ^3^Division of Genetics, Department of Pediatrics, New York University School of Medicine, New York, NY, USA; ^4^Division of Neonatology, Department of Pediatrics and Microbiology, New York University School of Medicine, New York, NY, USA

## Abstract

Congenital diaphragmatic hernia (CDH) in neonates may occur as an isolated finding, in association with other anomalies, or as part of a genetic syndrome. We report the first case of an infant with CDH who presented with hyponatremic seizures due to adrenal hypoplasia congenita (AHC). The patient underwent repair of CDH defect. After an uncomplicated postoperative course while on discharge planning, he developed a seizure episode associated with severe hyponatremia and hyperkalemia. Extensive diagnostic workup revealed an *NR0B1* gene variant confirming the diagnosis of X-linked AHC. The patient was eventually discharged home on hydrocortisone, fludrocortisone, and salt supplements. There are a few case reports of adrenal insufficiency in neonates with CDH, manifesting with symptoms before and immediately after reparative surgery. Clinical presentation of our patient was unique in manifesting as neonatal seizure secondary to severe hyponatremia after a stable postoperative phase. The patient's electrolytes and hemodynamic status remained stable before, during, and after surgery for CDH. This case underlines the importance of taking detailed family history and continued vigilance for signs and symptoms of adrenal insufficiency in infants with repaired CDH by pediatricians and intensivists.

## 1. Introduction

Congenital diaphragmatic hernia (CDH) in newborns is frequently associated with respiratory failure, persistent pulmonary hypertension, and catecholamine-resistant systemic hypotension. There is recently increased interest in understanding the potential role of adrenal insufficiency in catecholamine-resistant systemic hypotension in infants with CDH before and immediately after surgery [1]. Adrenal hypoplasia congenita (AHC), also known as congenital adrenal *hypo*plasia, is a disorder of adrenal development resulting in primary adrenal insufficiency. We report the first case of an infant with repaired CDH who presented with hyponatremic seizures due to AHC.

## 2. Case Presentation

An Asian male infant was born by vaginal delivery at 39 weeks of gestation, with a birth weight of 3410 g, to a 34-year-old mother (gravida: 3; parity: 1; preterm: 0; miscarriage: 1; liveborn: 1) with pregnancy notable for fetal diagnosis of left-sided CDH. Maternal obstetrical history was significant for an ectopic pregnancy and an uncomplicated birth of a healthy, term male infant. Family history was significant for a maternal uncle who died at 21 years of age and another maternal uncle and two maternal male cousins who died in early childhood, all due reportedly to adrenal issues. Given prenatal diagnosis and family history, both parents underwent Sema4 expanded carrier screening with no significant findings. Both parents screened negative for congenital adrenal hyperplasia. Amniocentesis revealed karyotype as 46,XY.

Physical examination at birth was notable for decreased breath sounds on the left side of the chest consistent with CDH, scaphoid abdomen, upslanting palpebral fissure, high-arched palate, smooth philtrum, and mild ankyloglossia. Skin exam was normal with no hyperpigmentation. Testes were descended bilaterally with a normal phallus.

Preoperative renal ultrasound was significant for mild bilateral hydronephrosis. Echocardiogram on day of life 2 revealed moderate pulmonary hypertension. He underwent uncomplicated repair of CDH on day of life 3. He remained hemodynamically stable before, during, and in the immediate postoperative period. He was extubated on day of life 8, successfully transitioned to room air by day of life 10, and reached full oral feeds by day of life 14. On day of life 17, he was noted to have decreased oral feeding and a brief episode of self-resolved bradycardia. A rule-out-sepsis workup was initiated. Soon after, the patient developed a prolonged generalized seizure, unresponsive to intravenous phenobarbital, and required emergent intubation.

**Figure 1:**
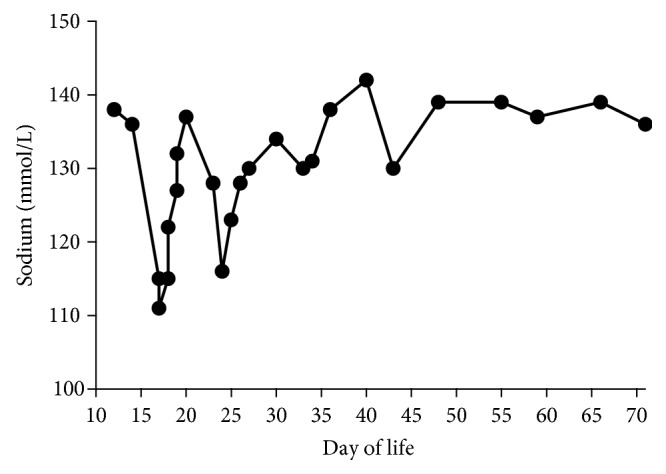
Serum sodium levels with days of life 10 through 70.

**Figure 2:**
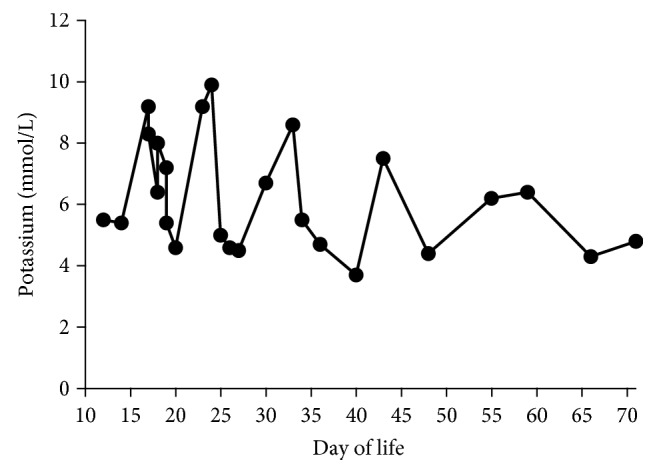
Serum potassium levels with days of life 10 through 70.

Initial laboratory results were notable for severe hyponatremia (Na^+^: 111 mmol/L) and hyperkalemia (K^+^: 9.2 mmol/L), a glucose level of 51 mg/dL, an arterial pH of 7.49, and a bicarbonate level of 16 mmol/L (reference range: 25–32 mmol/L) (Figures 1 and 2). He was treated with intravenous hypertonic saline, calcium gluconate, furosemide, an insulin infusion, dextrose 12.5% infusion, and albuterol nebulization. He was started on dopamine infusion secondary to systemic hypotension. Given this compilation of symptoms, adrenal insufficiency was suspected and hydrocortisone was initiated.

Over the next 48 hours, electrolyte derangements resolved. Blood, urine, and cerebrospinal fluid cultures were negative, and antibiotics were discontinued. Magnetic resonance imaging of the brain was normal. Initial video electroencephalogram was significant for epileptiform discharges but no seizures. An extensive endocrine evaluation was conducted. Some initial diagnostic labs were unable to be sent before the patient received hydrocortisone and hypertonic saline. Available labs were notable for low aldosterone (5.4 ng/dL, reference range: 7–99 ng/dL), normal dehydroepiandrosterone sulfate (87 *μ*g/dL, reference range: 32–431 *μ*g/dL), and normal 17-hydroxyprogesterone (159 ng/dL, reference range: <199 ng/dL). The sample for plasma renin was of insufficient quantity. The cortisol level was sent after starting hydrocortisone and thus deemed inconclusive.

Renal sonogram revealed a normal right adrenal and nonvisualized left adrenal gland. In view of the unconfirmed etiology and unrevealing testing by then, the baby was trialed off hydrocortisone on day of life 22, but hyponatremia and hyperkalemia recurred the following day with Na^+^ of 116 mmol/L and K^+^ of 9.9 mmol/L. The repeat cortisol level at this time was normal at 10.1 *μ*g/dL, and renin and aldosterone levels could not be obtained because of insufficient sample. Hydrocortisone was restarted, along with fludrocortisone and sodium chloride (NaCl) supplements. Enteral feeds were restarted with low mineral formula, Similac® PM 60/40. He had an additional episode of electrolyte derangement on day of life 43, which was managed by adjusting the dosage of hydrocortisone, fludrocortisone, and NaCl supplementation. Prior to discharge at 10 weeks of age, aldosterone remained low (3.8 ng/dL, reference range: 7–99 ng/dL), with normal renin levels (20.5 ng/ml/hr, reference range: 2.4–37 ng/mL/hr) on treatment. Newborn screening and microarray sent during admission were normal. He was subsequently discharged home on hydrocortisone, fludrocortisone, and NaCl supplements.

We considered the diagnosis of congenital adrenal hypoplasia (AHC) and requested DNA test of the *NR0B1* gene that is associated with AHC. Single gene sequencing and deletion/duplication analysis of the *NR0B1* gene (NM_000475.4) identified a variant of uncertain significance (VUS): c.1142T>C (p.Leu381Pro). This variant has never been reported before and is not present in population databases [2]. Different missense variants at this codon have been reported in individuals affected with X-linked AHC [3]. Missense pathogenic variants in *NR0B1* and the variant seen in our patient are located in the same protein domain [4]. These publications suggest that the leucine residue and the structural domain it resides may be critical for the *NR0B1* protein (DAX1) function [3, 4]. The mother was found to carry the same variant, which was absent in the 14-year-old unaffected brother. Therefore, this variant is likely clinically significant and is consistent with a diagnosis of X-linked AHC in our patient.

At 15 months of age, the patient is receiving physical and feeding therapy for oropharyngeal dysphagia. He is walking, has words, and is increasing his solid intake. He is currently on physiologic hydrocortisone, fludrocortisone, and NaCl supplementation. He has not had any electrolyte abnormalities since discharge from the hospital. He is followed every 2-3 months and will be monitored closely during adolescence for pubertal signs, as most children with AHC do not go into spontaneous puberty.

## 3. Discussion

CDH occurs in about 1 in 4000 newborns every year [5]. It can occur as an isolated finding, as part of a genetic syndrome (such as Cornelia de Lange and Fryns), as chromosomal abnormality (such as trisomies 18 and 21), or as a nonsyndromic set of findings [6]. Adrenal insufficiency may complicate the course of often-encountered catecholamine-resistant systemic hypotension in infants with CDH before corrective surgery and in a postoperative period of high stress [1]. To the best of our knowledge, this is the first case report of X-linked AHC in a patient with repaired CDH. Notably, the infant in our case did not show any signs and symptoms of adrenal insufficiency in the period of acute stress such as major surgery.

The differential for neonatal seizures is very diverse, including ischemic or hemorrhagic infarcts, meningitis, electrolyte derangements, and congenital anomalies or syndromes. Given our patient's clinical presentation and medical and family history, several diagnoses were considered including congenital adrenal hyperplasia, pseudoaldosteronism, and aldosterone synthase deficiency. Classic congenital adrenal hyperplasia was considered unlikely due to normal 17-hydroxyprogesterone level, normal newborn screens, and prenatal screening of the mother. Pseudoaldosteronism is typically associated with elevated aldosterone levels and thus was ruled out. In addition, aldosterone synthase deficiency type 1, where 18-hydroxycorticosterone is deficient with undetectable aldosterone levels, and type 2, where it is overproduced, with low to low-normal levels of aldosterone, were considered. One limitation we faced during evaluation was the inability to obtain steroid levels prior to the administration of glucocorticoids and mineralocorticoids.

Primary adrenal failure is a life-threatening condition that can be caused by a range of etiologies, including autoimmune, metabolic, and developmental disorders. The nuclear receptors DAX1 and steroidogenic factor 1 play an important role in adrenal and testicular development and function, and mutations in these transcription factors have been found in patients with adrenal hypoplasia [7, 8]. Adrenal insufficiency presents acutely, frequently in the neonatal period, with vomiting, hypoglycemia, feeding intolerance, and hypovolemic shock [9, 10].

About 1 in 12,500 newborn babies each year is born with AHC [7]. It results in primary adrenal insufficiency and has variable presentations. It can occur with several inheritance patterns such as X-linked AHC, with autosomal recessive forms, or as part of a syndrome. X-linked AHC accounts for half of the adrenal failure in boys not caused by congenital adrenal hyperplasia, autoimmune disease, or adrenoleukodystrophy [11]. It is caused by the mutations in the *NR0B1* gene that codes for the DAX1 nuclear transcription factor. Approximately half of the males present with AHC in early infancy. In others, the course is indolent, with progressive, chronic adrenal insufficiency. A typical lab presentation is hyponatremia, hyperkalemia, low aldosterone, low cortisol, and high adrenocorticotropic hormone. Interestingly, aldosterone deficiency has been found to precede cortisol deficiency, and some case series have described normal plasma cortisol at presentation, with decline over the first 6 months [12]. This may explain the relatively normal value on our random sample and the fact that the patient endured major surgery on day of life 3 without decompensation. The mainstay of medical management involves replacement of glucocorticoids and mineralocorticoids along with NaCl supplementation [13]. AHC may be associated with hypogonadotropic hypogonadism. Gonadotropins are the only pituitary deficits usually seen in the syndrome, as the production of other pituitary hormones is normal. Testosterone therapy may be required during adolescence for pubertal failure [12]. Female carriers are mostly unaffected. Therefore, a detailed family history, close monitoring, and a follow-up with genetics counselor are important for preventing life-threatening adrenal crises in close male relatives. In our patient, there was a remarkable family history of adrenal disorders with multiple close male relatives dying in early childhood, which helped in ultimately getting to the final diagnosis.

There have been reports of patients with genetically confirmed AHC whose presentations and laboratory abnormalities were consistent with congenital adrenal hyperplasia, highlighting the importance of confirmatory testing in cases of unexplained adrenal insufficiency in neonates [14]. While previous publications have described adrenal insufficiency in infants with CDH perioperatively [1], this patient exhibited no symptoms until his third week of life. In a patient population such as this, where feeding intolerance is not uncommon, it may prove prudent to check serum electrolytes and to take detailed family history during primary care visits.
